# Expression analysis of four pseudo-response regulator (PRR) genes in *Chrysanthemum morifolium* under different photoperiods

**DOI:** 10.7717/peerj.6420

**Published:** 2019-02-19

**Authors:** Shengji Wang, Chunlai Zhang, Jing Zhao, Renhua Li, Jinhui Lv

**Affiliations:** 1 College of Forestry, Shanxi Agricultural University, Jinzhong, China; 2 State Key Laboratory of Tree Genetics and Breeding, Northeast Forestry University, Harbin, China; 3 College of Agronomy, Shanxi Agricultural University, Jinzhong, China

**Keywords:** *Chrysanthemum*, Circadian clock, PRR, Flower bud differentiation, Gene expression

## Abstract

Genes encoding pseudo-response regulator (PRR) proteins play significant roles in plant circadian clocks. In this study, four genes related to flowering time were isolated from *Chrysanthemum morifolium*. Phylogenetic analysis showed that they are highly homologous to the counterparts of PRRs of *Helianthus annuus* and named as *CmPRR2*, *CmPRR7*, *CmPRR37*, and *CmPRR73*. Conserved motifs prediction indicated that most of the closely related members in the phylogenetic tree share common protein sequence motifs, suggesting functional similarities among the PRR proteins within the same subtree. In order to explore functions of the genes, we selected two *Chrysanthemum* varieties for comparison; that is, a short-day sensitive Zijiao and a short-day insensitive Aoyunbaixue. Compared to Aoyunbaixue, Zijiao needs 13 more days to complete the flower bud differentiation. Evidence from spatio-temporal gene expression patterns demonstrated that the *CmPRRs* are highly expressed in flower and stem tissues, with a growing trend across the *Chrysanthemum* developmental process. In addition, we also characterized the *CmPRRs* expression patterns and found that *CmPRRs* can maintain their circadian oscillation features to some extent under different photoperiod treatment conditions. These lines of evidence indicated that the four *CmPRRs* undergo circadian oscillation and possibly play roles in regulating the flowering time of *C. morifolium*.

## Introduction

Circadian clocks have evolved as a timekeeping molecular mechanism that enable organisms to predict and anticipate these periodic changes in their surrounding environment, for example, light/dark cycles and temperature oscillations (Nohales & Kay, 2016). The flowering time of plants is regulated by circadian clocks. In the photoperiodic response, light has two distinct functions: a resetting cue for circadian clock and a day-length signal. Light receptors can sense and transfer the light signal to biological circadian clock that impacts rhythmic outputs of downstream genes. These rhythm-regulated genes eventually activate or inhibit the expression of meristem genes and floral organs formation genes, which lead to the regulation of plant flowering. In *Arabidopsis*, the transcription and translation feedback loop, comprising the evening-expressed TIMING OF CAB EXPRESSION 1 (TOC1) gene, the morning-expressed CIRCADIAN CLOCK ASSOCIATED 1/LATE ELONGATED HYPOCOTYL (CCA1/LHY) gene and the evening complex EARLY FLOWERING 3 gene, is one of the core components of the oscillator (Alabadí et al., 2001; Hazen et al., 2005; Li et al., 2011; Perales & Más, 2007; Nusinow et al., 2012). Additional transcriptional repressors/co-repressors PSEUDO-RESPONSE REGULATORS 5 (PRR5), PRR7, and PRR9, TOPLESS, LUX ARRHYTHMO (LUX), and activators/co-activators REVEILLE (RVE) 4, RVE6, NIGHT LIGHT-INDUCIBLE AND CLOCK-REGULATED (LNK) 1, LNK2 are necessary in establishing the circadian clock (Cha et al., 2017; Farré et al., 2005; Kamioka et al., 2016; Hsu & Harmer, 2014).

Pseudo-response regulator represents a set of PRR proteins, which plays a significant role in circadian rhythm, light signal transduction, and flowering regulation. In *Arabidopsis*, many studies have reported the importance of PRR genes in the circadian clock (Eriksson et al., 2003; Farré et al., 2005; Ito et al., 2009; Kaczorowski & Quail, 2003; Michael et al., 2003; Norihito et al., 2005; Para et al., 2007; Prunedapaz et al., 2009; Salomé & Robertson McClung, 2005; Yoko et al., 2003). In *Arabidopsis*, the F box protein ZEITLUPE directly interacts with the pseudo-receiver (PR) domain of PRR5 and targets PRR5 for degradation by 26S proteasomes in the circadian clock and in early photomorphogenesis (Kiba et al., 2007). PRR5, PRR7, and PRR9 act as transcriptional repressors of CCA1 and LHY. The repressor activities of these proteins account for their roles in the interlocking feedback loop of the circadian clocks (Nakamichi et al., 2010). TOC1 can repress direct targets through the CCT motif, and the repression activity is in the PR domain of the protein (Gendron et al., 2012). PRR5, through binding to the CCT motifs of its target genes, is involved in flowering-time regulation, hypocotyl elongation and cold-stress (Nakamichi et al., 2012). Evidence from reverse genetics indicated that several other *PRR* genes also have the function of changing the length of the circadian cycle (McClung, 2006). *SbPRR37* can inhibit the flowering process of *Sorghum* under the long sunshine condition, with its expression level influenced by the photoperiod (Murphy et al., 2011). *PRR37* down-regulates *Hd3a* expression to suppress rice flowering under long-day conditions (Koo et al., 2013). In long-day plants such as barley and wheat, *AtPRR7* homologous gene *PHOTOPERIOD 1* plays a critical role in photoperiod response and flowering regulation (Boden et al., 2015; Turner et al., 2005). *OsPRR73*, a rice *PRR* gene co-expressed with Hdl protein gene *OsBBX29*, is involved in regulating the flowering process through nucleic acid metabolism pathway (Iniesta et al., 2008; Murakami et al., 2007).

Similarly to the PRRs, GIGANTEA (GI), a large plant-specific protein family, which lacks well-characterized functional domains, is also required for circadian timekeeping (Gould et al., 2006; Martin-Tryon, Kreps & Harmer, 2007). GI is involved not only in the central oscillator but also in light input and photoperiodic flowering output pathways (Gould et al., 2006; Martin-Tryon, Kreps & Harmer, 2007; Mishra & Panigrahi, 2015). *GI* genes can be repressed in the morning by CCA1 and LHY (Lu et al., 2012), both of which in turn appear to be induced by GI (Martin-Tryon, Kreps & Harmer, 2007). In addition, TOC1 and EC (evening complex) both contribute to *GI* repression (Huang et al., 2012; Mizuno et al., 2014).

*Chrysanthemum morifolium*, one of the most important global cut-flowers and pot plants, is a typical short-day plant that is distributed worldwide (Teixeira da Silva, 2003; Liu et al., 2016). Breeders have attempted to develop more *Chrysanthemum* varieties with different flowering times and colors which promote *C. morifolium* as an ideal material to study the light inducible flowering system (Teixeira da Silva, 2003; Liu et al., 2016). Several flowering-related genes, such as *COL1*, *COL5*, *ClFT1*, and *ClFT2,* have been cloned from *C. lavandulifolium* and transferred into *Arabidopsis* (Fu, 2014). For instance, research indicated that transgenic *Arabidopsis* overexpressing *COL1* and *COL5* are both bolting and flowering earlier than wild-type strains. *ClFT1* and *ClFT2* may play opposite roles in the flowering process, as *ClFT1* transgenic *Arabidopsis* is flowering earlier than the control, while *ClFT2* transgenic *lines* are flowering later compared to the control (Fu, 2014). Transgenic *Chrysanthemum* with overexpression of *CsFTL3* is able to bloom normally even under long-day conditions (Atsushi et al., 2012). However, there are limited studies on the *PRR* genes of *C. morifolium*. In this study, we compared the flower bud differentiation process between short-day sensitive *C. morifolium* varieties Zijiao and short-day insensitive Aoyunbaixue and found that Zijiao needs 13 more days than Aoyunbaixue to complete the flower bud differentiation. We then cloned four putative PRR genes (*CmPRR2*, *CmPRR7*, *CmPRR37*, and *CmPRR73*) from *C. morifolium*, and showed their phylogenetic relationship related to PRRs from *Arabidopsis* and other species. Expression patterns of these four CmPRR genes under different photoperiod conditions and across different flower bud differentiation stages were also analyzed to understand their functions in regulating flowering time or circadian rhythms of *C. morifolium*.

## Materials and Methods

### Cultivation of *Chrysanthemum morifolium* variety

The short-day sensitive *C. morifolium* variety Zijiao and insensitive variety Aoyunbaixue were cultivated in the greenhouse of Shanxi Agricultural University under normal growth conditions with 60–70% relative humidity, 14/10-h light/dark cycles, and a constant temperature of 25 ± 2 °C.

### Gene cloning and phylogenetic analysis

Tender leaves were harvested from Aoyunbaixue, frozen immediately in liquid nitrogen, and stored at −70 °C for RNA isolation and reverse transcription-polymerase chain reaction (RT-PCR). Primers targeting the UTR (Untranslated Region) region were designed and used to amplify the coding sequences of *CmPRR2*, *CmPRR7*, *CmPRR37*, and *CmPRR73* according to the RNA-seq data (Table 1). RT-PCR products were then purified and sent to BGI Shenzhen (Shenzhen, China) for DNA sanger sequencing to confirm the identity of the fragments. Non-reverse transcribed RNA was used as the negative control to judge amplified DNA is from mRNA not genomic DNA.

**Table 1:** Primers of *Chrysanthemum PRRs* used in gene cloning.

Genes	Primers (5′-3′)		cDNA length (bp)
	Forward	Reverse	
*PRR2*	AGCTATGGTTTGCACTGCGAAC	CAGCATTAACGAGAGCTGCTGAT	1,605
*PRR7*	GTTGATGAGGAGTGTTGGTGT	CAAGAGCTCTGAGTTCCACTTC	1,017
*PRR37*	GGTTAATGAAGAGTGTTGGAGTG	TCAGTAGTCCGACAAACCGA	2,019
*PRR73*	TCGATGACTAGTAGCAGCAGAG	ACTAGACATCAGCAGCATTAGCG	1,941

Nine PRR protein sequences from *Arabidopsis* and 18 from other related species were blasted and downloaded from the NCBI (https://www.ncbi.nlm.nih.gov/gene/). Multiple sequence alignments of PRR proteins were performed using Clustal X 1.83 (Jeanmougin et al., 1998). Unrooted phylogenetic trees were constructed with MEGA 7.0.21 using the neighbor joining (NJ) methods and the bootstrap test carried out with 1,000 iterations (Kumar, Stecher & Tamura, 2016). Pairwise gap deletion mode was used to ensure the divergent domains could contribute to the topology of the NJ tree (Kumar, Stecher & Tamura, 2016). Conserved motifs in PRR proteins were also detected using the program MEME version 5.0.2 (Bailey et al., 2009). MEME was run with the following parameters: any number of repetitions, 10 maximum motifs, and between 6 and 50 residues for the optimum motif widths.

### RNA extraction and RTq-PCR analysis

Total RNA of each sample material was extracted using the Column Plant RNAout Kit (Tiandz, Beijing, China) followed by cDNA synthesis using PrimeScipt^™^RT reagent Kit (Takara, Dalian, China) according to the manufacturer’s instructions. Quality and quantity of RNA were determined by agarose gel electrophoresis and a NanoDrop 2000c Spectrophotometer (NanoDrop Technologies, Wilmington, DE, USA). RTq-PCR was performed on an ABI 7,500 real-time PCR system (Applied Biosystems, Foster City, CA, USA) using the SYBR Premix Ex Taq^™^ kit (Takara, Dalian, China) according to the manufacture’s instructions. Primers used for RTq-PCR are shown in Table 2. The amplification curve was generated after analyzing the raw data and the cycle threshold value was calculated based on the fluorescence threshold of 0.01 (Bustin et al., 2009; Gu et al., 2011; Bustin & Mueller, 2005). The average expression levels of *Chrysanthemum* house-keeping genes *Actin* and *UBC* were used as the internal control (Table 2). The relative expression level of target genes in different samples was calculated using 2^−ΔΔCt^ method, defined as ΔΔC_*t*_ = (C_*t*-target_—C_*t*-control_)_2_–(C_*t*-target_—C_*t*-control_)_1_ (Gu et al., 2011; Bustin & Mueller, 2005). RTq-PCR was carried out with three technical and three biological replicates per sample.

**Table 2:** Primers used in RTq-PCR.

Genes	Primers (5′-3′)	
	Forward	Reverse
*Actin*	CCAAAAGCCAATCGTGAGAAG	CACCATCACCAGAATCCAACA
*UBC*	TCTCGCTTGTCCGGTTTGTG	ACCTTGGGTGGCTTGAATGG
*CmPRR2*	GTGAGGGCAGACAACGAAGA	TTCTCGAGGAATTCGACCGC
*CmPRR73*	GGGATGACGATGAGAGCACC	ACGGATAACGAGGCCACAAG
*CmPRR7*	TTGTTCAGTGGGAGCGGTTT	ATAGCCGCAATTACGGAGCA
*CmPRR37*	AGCATCCTTCCCATTCACCC	CAAGTTCCGCCAGAAGAGGA
*CmCOL1*	GTGTCCCGGTTATGCCTATTTC	CGCTGCTTCGTCTTCTTCTTC
*CmCOL5*	GTTCTTGTGTCTCGCGTGTG	AGCATCAGCCTTACACGTCA
*COL9Y*	TTGGTGGTGCCGAATCTTCA	CCTTCCATAGCGGGTTGGTT
*CmCOL13*	TACCCAAGAACGGGAGACCG	GCAAAGCGACCTCTGATCCT
*COL14Y*	ACAGCTAACGTGAGCAGCAT	TTCTGCAACAATAGGCCAGC
*CmCOL15*	TGAAACCGTCGACAGAGGTG	GTCGTTCTGCTCCTGTCTCC
*COL16Y*	CCACGACCAAGGAGCAAATAC	ACACGGGCACTAAAGGATACAAA
*CmCOL20*	TGTCCAGCTGACGATGCTTT	GAAACTGCGCCTGAACCAAA
*CmGI1-F*	ATGGATAGCGGTGACGAACC	GCCTCCCCCATTAGATACGC
*CmGI2-F*	GCGAAAATACCGATGCCACC	TAGCTGAAGTTCGCAGGCAA
*CmGI3-F*	GAGTTGGTTCACCACCGCTA	ACCAGCGGAAGTAGTCATGC
*CmFT-F*	ACAGGAGCACAGTTTGGTCA	ACCCAATTGCCGGAATAGCA

### Expression patterns of CmPRRs across different flower bud differentiation stages of Zijiao and Aoyunbaixue

In the early stage of vegetative growth, mature leaves at the 3rd and 4th nodes were harvested every 3 days at 9:00 am from respective Zijiao and Aoyunbaixue, in order to study the expression patterns of *CmPRR2*, *CmPRR7*, *CmPRR37*, and *CmPRR73* across nine different flower bud differentiation stages (Vegetative growth stage, Inflorescence primordial differentiation stage, Initial stage of involucres primordial differentiation, Final stage of involucres differentiation, Initial stage of ligulae flower differentiation, Final stage of ligulae flower differentiation, Initial stage of corolla formation, Secondary stage of corolla formation, and Finish stage of corolla formation). For comparison, other genes related to circadian clock, including *CmGI1*, *CmGI2*, *CmGI3*, *CmCOL1*, *CmCOL5*, *CmCOL9*, *CmCOL13*, *CmCOL14*, *CmCOL15*, *CmCOL16*, *CmCOL20*, and *CmFT*, were also used in the study (Table 2). All of the leaf samples were stored at −70 °C for RNA isolation and RTq-PCR analysis. Terminal buds of Zijiao and Aoyunbaixue were cut every 3 days in addition to the leaf tissue harvested. Then the bud materials were fixed with FAA (Formalin–acetic acid–alcohol) solution for 1 week and used for paraffin section operation to observe the flower bud differentiation process under the microscope (Gerlach, 1969).

### Expression pattern of CmPRRs in Zijiao across different developmental stages

Mature leaves at the 3rd and 4th nodes were harvested at 9:00 am from Zijiao at the respective stages of flower bud undifferentiation, squaring, visible bud color, initial flowering, full-blossom, and the senescing flowers. Materials were collected and stored at −70 °C for RNA isolation and gene expression analysis using RTq-PCR.

### Expression patterns of CmPRRs in Zijiao different tissues

Since *CmPRRs* are highly expressed at the senescing flower stage (will be addressed below), we harvested roots, stems, leaves, and flowers from Zijiao at 9:00 am at this stage, in order to study the tissue-specific expression of the *CmPRRs* using RTq-PCR.

### Expression patterns of CmPRRs in Zijiao under different photoperiod conditions

#### Long- or short-day treatments

Stem cuttings from Zijiao were propagated in the green house with 60–70% relative humidity, 14/10-h light/dark cycles and a constant temperature of 25 ± 2 °C. 1 month later, at least 50 strong and healthy seedlings were selected and planted in flowerpots for long-day (LD; 16/8-h light/dark cycles) and short-day (SD; 8/16-h light/dark cycles) treatments, respectively. After 2 weeks, leaves at the 3rd and 4th nodes were harvested every 3 h within 24 h for gene expression analysis using RTq-PCR. Materials collection was carried out with three biological replications at every time point.

#### Night-break treatment

After 2 weeks of short-day treatment, seedlings were further treated with night-break; that is, light was on at 15 h, was off at 17 h, and lasted for 2 h. After that, leaves at the 3rd and 4th nodes were harvested every 3 h within 24 h for gene expression pattern analysis. Materials collection was carried out with three biological replications at every time point.

#### Continuous light or dark treatment

After being treated with the long-day or short-day photoperiod for 2 weeks, seedlings were transferred to continuous light or dark conditions. Then leaves at the 3rd and 4th nodes were harvested every 3 h within 48 h for gene expression analysis. Period length and relative amplitude were estimated using program FFT-NLLS (Millar et al., 1995; Plautz et al., 1997). Materials collection was carried out with three biological replications at every time point.

#### Statistical analysis

Statistical analysis methods included single variable analysis and multiple variable analysis. Single variable analysis was used to compare the *CmPRRs* gene expression in Zijiao across different development stages (FU, SQ, VB, IF, FB, SF), and to compare gene expression level in Zijiao in different tissues (Flowers, Leaves, Stems, Roots). The student *t-*test was applied for this purpose. Multivariate analysis was employed to study the relationships and interactions between the two variables: different *Chrysanthemum varieties* (Zijiao and Aoyunbaixue) and different flower bud differentiation stages. The multiple linear regression modeling followed by the analysis of variance was performed to examine the significance of one variable after adjustment for another variable. In addition, the errors from both technical and biological replications were illustrated using standard error (SE) method. Data represents the mean ± SE of the experiments. All statistical analyses were conducted using the package corrplot in R (R Core Team, 2018).

## Results

### Gene cloning and amino acids sequence analysis

**Figure 1:**
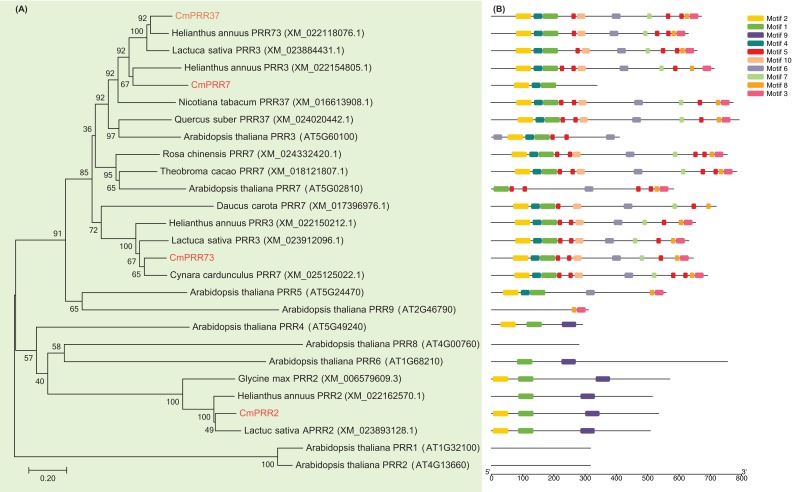
Phylogenetic relationships and motif composition of PRRs. (A) Multiple alignment of full-length amino acids of PRRs. (B) Schematic representation of conserved motifs in the PRR proteins.

The cDNA fragments of *CmPRR2*, *CmPRR7*, *CmPRR37*, and *CmPRR73* were amplified using RT-PCR from *Chrysanthemum* Aoyunbaixue according to the RNA sequencing pre-test (SI.1, SI.2). In order to examine the phylogenetic relationships among the PRR protein sequences from *Chrysanthemum* and other related species, we constructed unrooted trees based on alignments with the full-length protein sequences. Evidences from the sequence comparisons indicated that the *Chrysanthemum* PRRs are highly homologous to the counterparts from other species, especially the *Asteraceae* plant *Helianthus annuus* (Fig. 1A). For instance, CmPRR2 shares high homology with the PRR2 from *H. annuus* (XM_022162570.1), *Glycine max* (XM_006579609.3), and *Lactuca sativa* (XM_023893128.1) (Fig. 1A). CmPRR7 has high similarity with protein TaPRR7 from *Theobroma cacao* (XM_018121807.1), HaPRR3 from *H. annuus* (XM_022154805.1), and NtPRR37 from *Nicotiara tabacum* (XM_016613908.1). CmPRR37 shares 82%, 83%, and 77% sequence identity with protein HaPRR73 (XM_022118076.1), LsPRR3 (XM_023884431.1), and QsPRR37 (XM_024020442.1), respectively. CmPRR73 also shows high similarity to HaPRR3 (XM_022150212.1) and LsPRR3 (XM_023912096.1). However, we found that all of the four CmPRR proteins have a relatively low homology with PRR proteins from *Arabidopsis* (Fig. 1A).

To further reveal the diversification of the PRR genes, putative motifs were predicted by the program MEME, and 10 distinct motifs were identified (Fig. 1B). Details of the 10 putative motifs are shown in Supplementary Information 3. Most of the closely related members in the phylogenetic tree have common motif compositions, suggesting functional similarities among the PRR proteins within the same subtree (Fig. 1). All of the CmPRR2, CmPRR7, CmPRR37, and CmPRR73 contain the conserved Motif1 and Motif2 in the N-terminal of amino acid sequence (Fig. 1B). Moreover, CmPRR37 and CmPRR73 share high consistency in the motifs harbored (Fig. 1B).

### Observation of flower bud differentiation process

**Figure 2:**
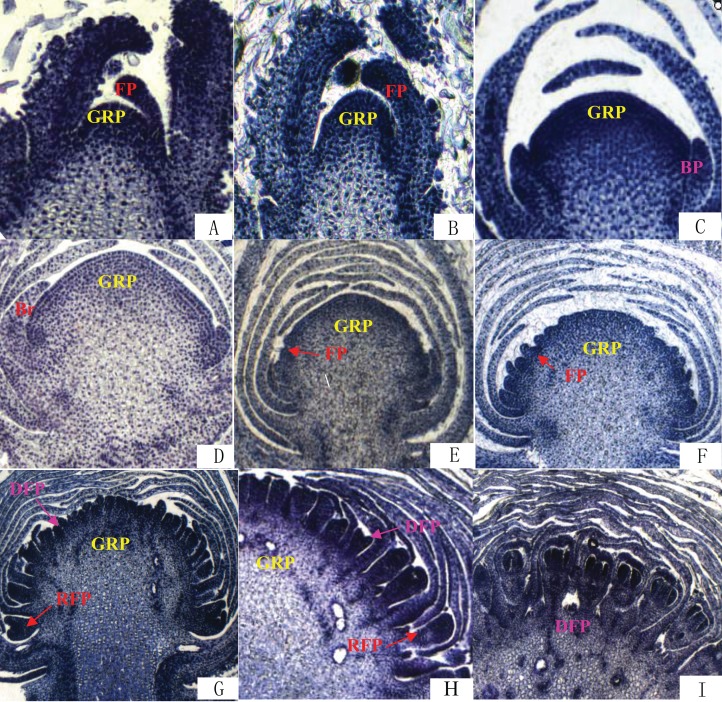
Flower bud differentiation observation using paraffin section. (A) Vegetative growth stage; (B) inflorescence primordial differentiation stage; (C) initial stage of involucres primordial differentiation stage; (D) final stage of involucres differentiation; (E) initial stage of ligulae flower differentiation; (F) final stage of ligulae flower differentiation; (G) initial stage of corolla formation; (H) secondary stage of corolla formation; (I) finish stage of corolla formation; Br, Bract; BP, Bract primordium; DFP, Disk primordium; FP, Floret primordium; GRP, Growth point; RFP, Ray floret primordium. For paraffin section operation to observe the flower bud differentiation process under the fluorescence microscope. The paraffin section operation was performed with about 10 buds per samples.

The differentiation of *Chrysanthemum* buds is divided into two processes: inflorescence differentiation and floret differentiation. The former contains such stages as vegetative growth, inflorescence primordial differentiation, involucres differentiation, and ligulae flower differentiation. The latter mainly has the corolla formation stage. On the basis of the results of paraffin section (Fig. 2), we compared the time of flower bud differentiation between Zijiao and Aoyunbaixue (Table 3). The flower bud of Zijiao started to differentiate after 9 days of vegetative growth, and floret differentiate initiated after 54 days of vegetative growth (Table 3). The entire flowering process lasted for 64 days. In contrast, Aoyunbaixue flower bud differentiation started after 15 days of vegetative growth, and floret differentiate initiated after 39 days of vegetative growth (Table 3). The entire flowering process took 51 days. Compared to Aoyunbaixue, Zijiao needs 13 more days to complete the flower bud differentiation. In addition, the initiation of flower bud differentiation in Zijiao is 38 days later compared to that of Aoyunbaixue.

**Table 3:** Compare of flower bud differentiation times between *Chrysanthemum* Zijiao and Aoyunbaixue.

Stage	Start time		Last time (Days)	
	Zijiao	Aoyunbaixue	Zijiao	Aoyunbaixue
A-Vegetative growth stage	14, June	30, April	9	15
B-Inflorescence primordial differentiation stage	23, June	15, May	18	10
C1-Initial stage of involucres primordial differentiation stage	11, July	25, May	21	10
C2-Final stage of involucres differentiation	2, August	4, June	6	4
D1-Initial stage of ligulae flower differentiation	8, August	8, June	3	7
D2-Final stage of ligulae flower differentiation	11, August	15, June	3	7
E1-Initial stage of corolla formation	14, August	22, June	6	3
E2-Secondary stage of corolla formation	20, August	25, June	6	15
E3-Finish stage of corolla formation	26, August	9, July		

### Expression patterns of CmPRRs in Zijiao and Aoyunbaixue flower bud differentiation stages

**Figure 3:**
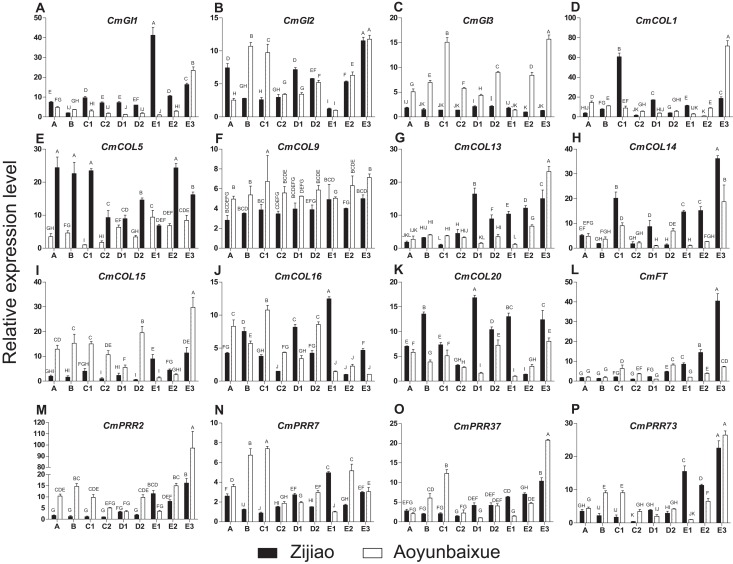
Expression pattern of circadian clock related genes across Zijiao and Aoyunbaixue different flower bud differentiation stages. A-Vegetative growth stage; B-Inflorescence primordial differentiation stage; C1-Initial stage of involucres primordial differentiation stage; C2-Final stage of involucres differentiation; D1-Initial stage of ligulae flower differentiation; D2-Final stage of ligulae flower differentiation; E1-Initial stage of corolla formation; E2-Secondary stage of corolla formation; E3-Finish stage of corolla formation. Capital letters (A–P) indicate whether the samples are significantly different from each other. Data represents the mean ± SE of three technical and three biological repeats per sample.

We compared gene expression patterns between short-day sensitive Zijiao and short-day insensitive Aoyunbaixue. Four genes, including *CmPRR2*, *CmPRR7*, *CmPRR37*, and *CmPRR73,* were overexpressed in Aoyunbaixue at stage E3 compared to Zijiao (Fig. 3). Before the E3 stage, expression levels of *CmPRR2* were relatively low in both Zijiao and Aoyunbaixue (Fig. 3). The expression of *CmPRR7* was significantly different between Zijiao and Aoyunbaixue across the entire flower bud differentiation stages except the E3 stage. It reached to the peak at stage D1 in Aoyunbaixue, while in Zijiao the peak displayed at stage E1 (Fig. 3). *CmPRR37* and *CmPRR73* showed similar gene expression patterns, except that their expression levels were relatively higher in Zijiao than that in Aoyunbaixue at stages D1, D2, E1, and E2, and vice versa at the other stages (Fig. 3). In addition, expression levels of other circadian clock related genes, including *CmGI1*, *CmGI2*, *CmGI3*, *CmCOL1*, *CmCOL5*, *CmCOL9*, *CmCOL13*, *CmCOL14*, *CmCOL15*, *CmCOL16*, *CmCOL20*, and *CmFT*, were identified using RTq-PCR. In general, *CmCOL14*, *CmCOL20*, and *CmFT* genes showed opposite expression patterns with the *CmPRRs* gene at stage E3. However, genes, including*CmGI1, CmGI3, CmCOL1, CmCOL5, CmCOL13*, and *CmCOL15,* displayed a similar expression pattern with the *CmPRRs* (Fig. 3). In addition, there was no significant difference between Zijiao and Aoyunbaixue in the expression of *CmCOL9* during the flower bud differentiation.

### Expression patterns of CmPRRs in Zijiao across different developmental stages

**Figure 4:**
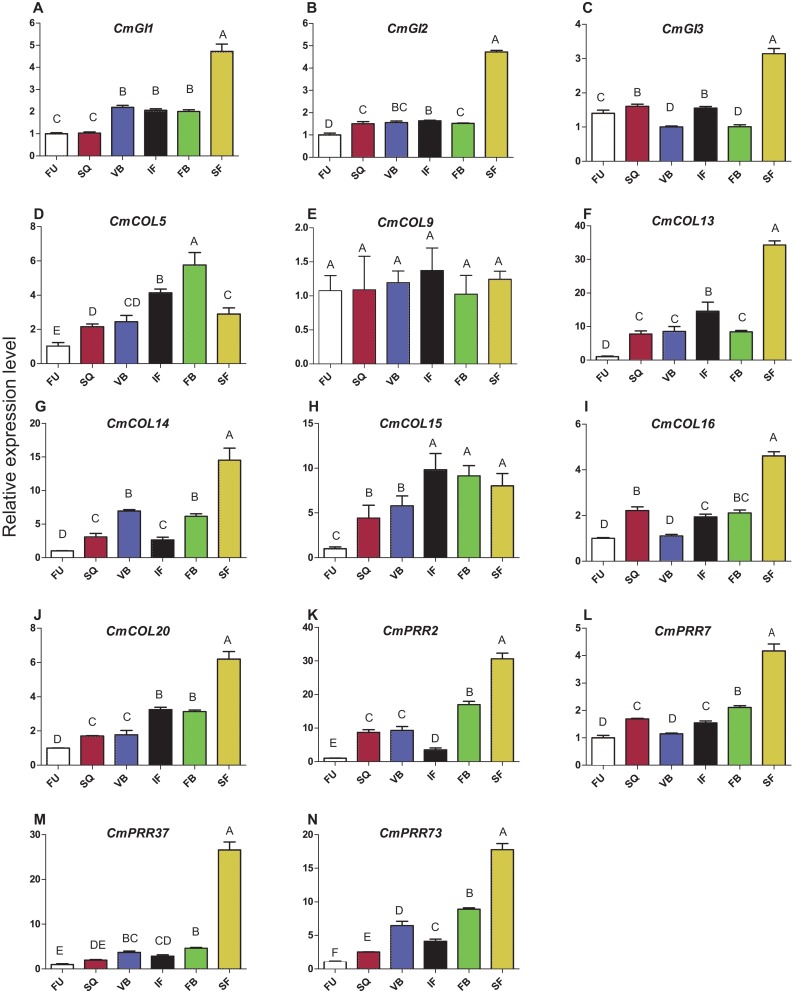
Expression pattern of circadian clock genes in Zijiao different development stages. (A) Relative expression level of *CmGI1*; (B) Relative expression level of *CmGI2*; (C) Relative expression level of *CmGI3*; (D) Relative expression level of *CmCOL5*; (E) Relative expression level of *CmCOL9*; (F) Relative expression level of *CmCOL13*; (G) Relative expression level of *CmCOL14*; (H) Relative expression level of *CmCOL15*; (I) Relative expression level of *CmCOL16*; (J) Relative expression level of *CmCOL20*; (K) Relative expression level of *CmPRR2*; (L) Relative expression level of *CmPRR7*; (M) Relative expression level of *CmPRR37*; (N) Relative expression level of *CmPRR73*. AFU, Flower bud undifferentiated stage; SQ, squaring stage; VB, visible bud color stage; IF, initial flowering stage; FB, full blossom stage; SF, senescing flower stage. Letters in (A–N) indicate whether the samples are significantly different from each other. Data represents the average ± SE of three technical and three biological repeats per sample.

Expression patterns of circadian clock genes, including *CmPRR2*, *CmPRR7*, *CmPRR37*, and *CmPRR73*, were analyzed across developmental stages of Zijiao (Fig. 4). In general, expression levels of *CmPRR2*, *CmPRR7*, *CmPRR37*, and *CmPRR73* showed a growing trend across *Chrysanthemum* developmental process. The expression of *CmPRR2* and *CmPRR73* showed a brief decrease at the initial flowering stage; in contrast, the expression of *CmPRR7* went down at the stage of visible bud color (Fig. 4). At squaring stage, the expression level of *CmPRR2* is almost five times that of the other three *CmPRRs* (Fig. 4). At the visible bud color stage, the expression level of *CmPRR73* was twice that of *CmPRR37*, and expression level of *CmPRR37* was three times that of *CmPRR7* (Fig. 4). However, other genes, including *CmGI1* and *CmCOL20,* exhibited a gradual increase in expression across the entire developmental stages of Zijiao, which was consistent with the expression of *CmPRR37*. In addition, the expression patterns of *CmGI2*, *CmGI3*, *CmCOL13*, *CmCOL14*, and *CmCOL16* were similar to that of *CmPRR2*, *CmPRR7*, and *CmPRR73* (Fig. 4).

### Expression patterns of CmPRRs in different tissues of Zijiao

**Figure 5:**
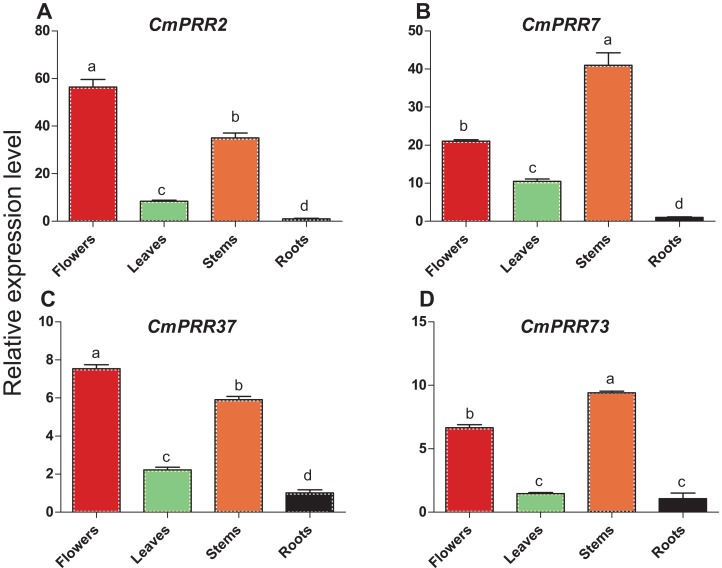
Expression pattern of *CmPRRs* in different tissues of Zijiao. (A) Relative expression level of *CmPRR2*; (B) Relative expression level of *CmPRR7*; (C) Relative expression level of *CmPRR37*; (D) Relative expression level of *CmPRR73*. Letters a–d indicate whether the samples are significantly different from each other. Data represents the average ± SE of three technical and three biological repeats per sample.

Expression patterns of *CmPRR2*, *CmPRR7*, *CmPRR37*, and *CmPRR73* in the respective root, stem, leave, and flower tissues of Zijiao were analyzed. These four genes were expressed in all of the tissues tested, with high expression level in flowers and stems, and the lowest level in roots (Fig. 5). *CmPRR2* and *CmPRR37* displayed similar expression patterns to that of *CmPRR7* and *CmPRR73*. It is notable that the expression of *CmPRR7* in roots was significantly lower than that in the leaf; but the expression of *CmPRR73* in roots was no significantly different from that in leaves.

### Expression patterns of CmPRRs in Zijiao under different photoperiod conditions

#### Long- or short-day treatments

**Figure 6:**
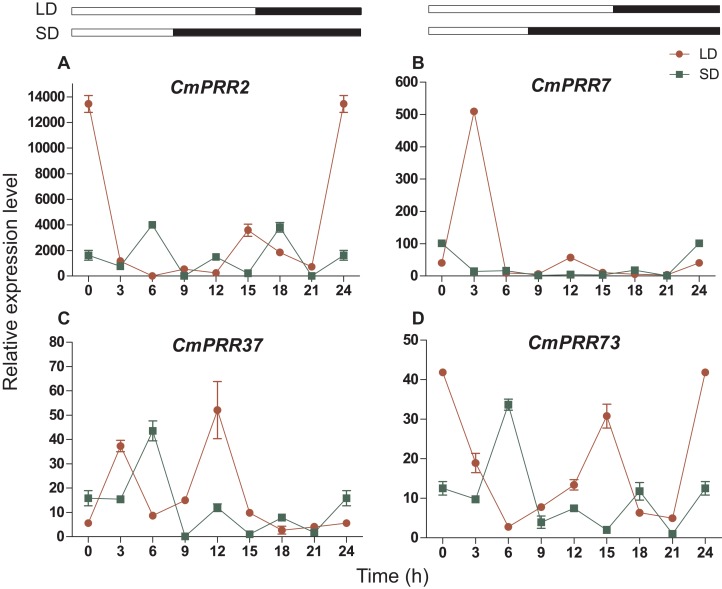
Expression pattern of *CmPRRs* of Zijiao in long-day and short-day photoperiod conditions. (A) Relative expression level of *CmPRR2*; (B) Relative expression level of *CmPRR7*; (C) Relative expression level of *CmPRR37*; (D) Relative expression level of *CmPRR73*. LD, Long-day condition; SD, short-day condition. Data represents the average ± SE of three technical and three biological repeats per sample.

The *CmPRR* gene responded differentially to long- and short-day treatments. Expression of *CmPRR2* was similar to that of *CmPRR73* under both long- and short-day conditions; that is, the expression peaks of the two genes coincide with one another, and their expression levels were higher under long-day condition, compared to the short-day condition (Fig. 6). When challenged with long-day, both *CmPRR2* and *CmPRR73* expressed the highest level at the start of light and the end of darkness; In contrast, under the short-day condition, the peak of expression occurred at 6 h in the light phase. Under the long-day condition, expression level of *CmPRR7* started to increase upon the light, reached to the peak after 3 h, and then declined (Fig. 6). Under short-day condition, however, its expression generally continued to decline upon light (Fig. 6). Nevertheless, expression of *CmPRR7* showed a rebound at the end of dark phase in both long- and short-day conditions. Compared to the dark phase*, CmPRR37* expressed at a higher level in the light phase under both long- and short-day conditions, with the peak occurred before the end of light treatment (Fig. 6).

#### Night-break treatment

**Figure 7:**
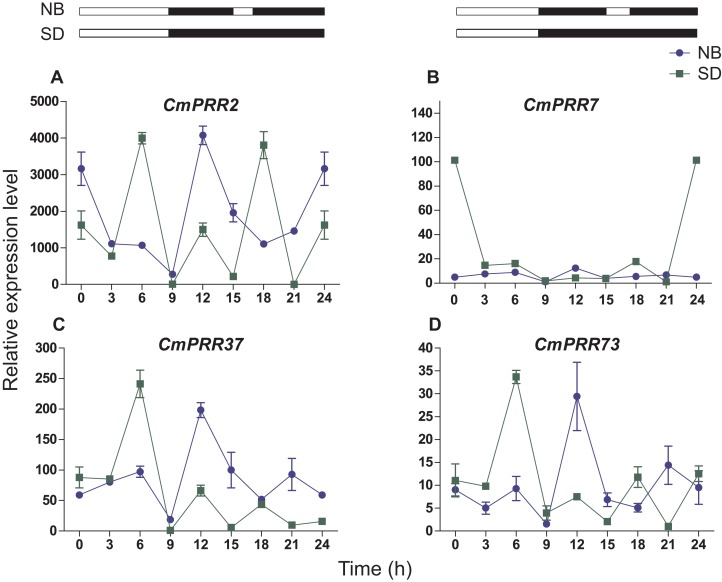
Expression pattern of *CmPRRs* of Zijiao in night-break condition. (A) Relative expression level of *CmPRR2*; (B) Relative expression level of *CmPRR7*; (C) Relative expression level of *CmPRR37*; (D) Relative expression level of *CmPRR73*. The light break occurred at 15 h, went off at 17 h, and lasted for 2 h. NB, Night-break condition; SD, short-day condition. Data represents the average ± SE of three technical and three biological repeats per sample.

Under the night-break condition, expression levels of *CmPRR2*, *CmPRR37*, and *CmPRR73* were similar to that challenged with the short-day, with the peak of expression levels coincided with one another (Fig. 7). In contrast, expression level of *CmPRR7* was significantly reduced due to the night-break, compared to its expression under the short-day condition. When challenged with the night-break, the expression of *CmPRR2*, *CmPRR37*, and *CmPRR73* genes decreased at 15–17 h, which was the opposite to that with the short-day treatment (Fig. 7). After 3-h night-break, the expression levels of *CmPRR2*, *CmPRR37*, and *CmPRR73* started to rise, but still displayed a trend that was against that under the short-day condition (Fig. 7). In addition, *CmPRR7* gene expression level was relatively stable at 15–17 h (Fig. 7).

#### Continuous light

**Figure 8:**
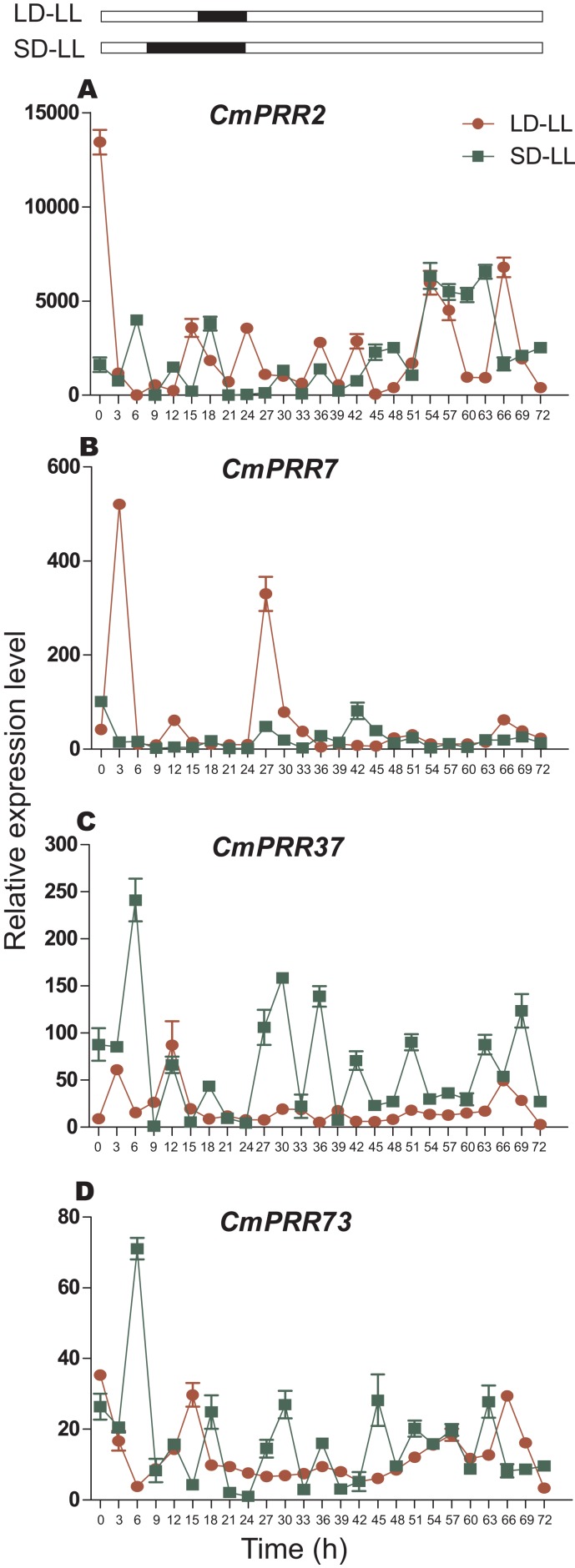
Expression pattern of *CmPRRs* of Zijiao in continuous light condition. (A) Relative expression level of *CmPRR2*; (B) Relative expression level of *CmPRR7*; (C) Relative expression level of *CmPRR37*; (D) Relative expression level of *CmPRR73*. LD, Long-day condition; SD, short-day condition; LL, continuous light condition. Data represents the average ± SE of three technical and three biological repeats per sample.

After switching from the long-day to the continuous light condition, expression of *CmPRR2* and *CmPRR7* still maintained the original circadian rhythm in the first 24 h, but the expression amplitude decreased (Fig. 8). Similarly, *CmPRR37* and *CmPRR73* also maintained a weak circadian rhythm. In the following 24 h, the four *CmPRRs* maintained a varied circadian rhythm (Fig. 8). After changing from the short-day to the continuous light condition, expression of *CmPRR37* still kept the original circadian rhythm with the amplitude decreased in the first day (Fig. 8). Expression of *CmPRR7* had a weak circadian rhythm (Fig. 8). *CmPRR2* and *CmPRR73* showed similar expression patterns with that under the long-day condition. In the following day, *CmPRR7* hold a certain circadian rhythm, with a reduced amplitude.

#### Continuous dark

**Figure 9:**
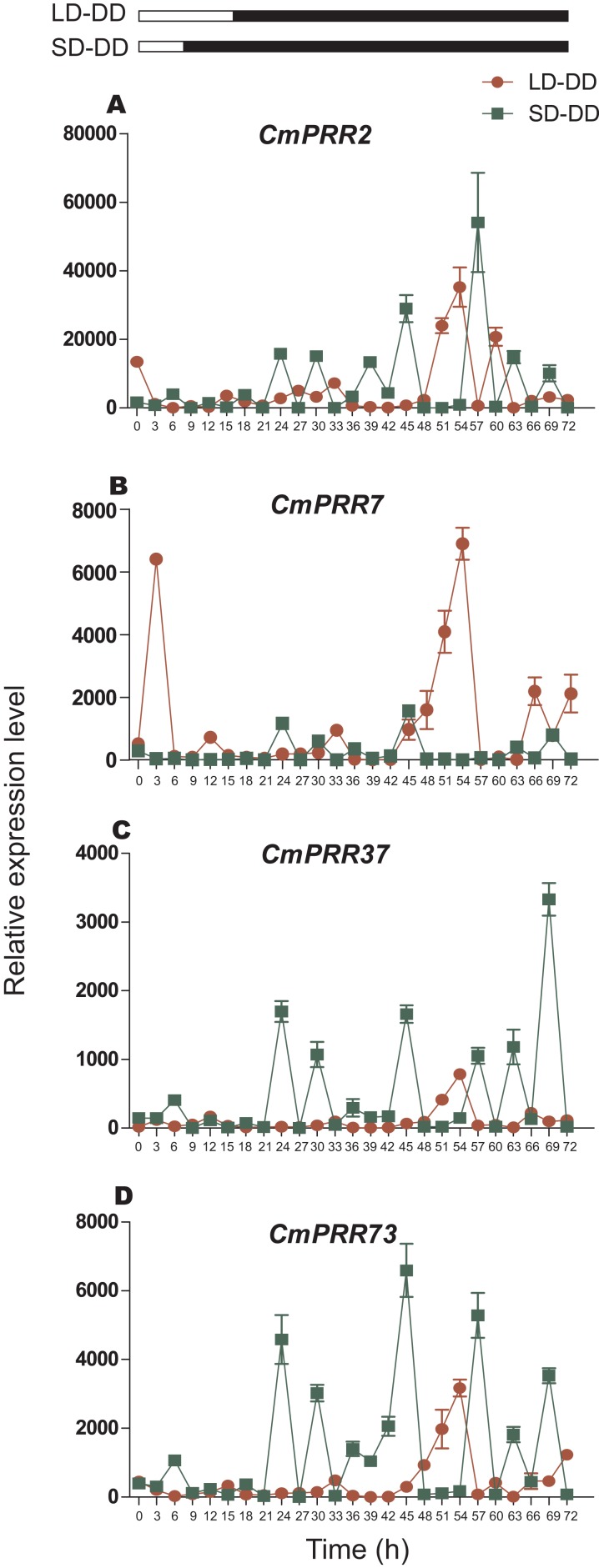
Expression pattern of *CmPRRs* of Zijiao in continuous dark condition. (A) Relative expression level of *CmPRR2*; (B) Relative expression level of *CmPRR7*; (C) Relative expression level of *CmPRR37*; (D) Relative expression level of *CmPRR73*. LD, Long-day condition; SD, short-day condition; DD, continuous dark condition. Data represents the average ± SE of three technical and three biological repeats per sample.

After switching from the long-day to the continuous dark condition, expression of *CmPRR7*, *CmPRR37*, and *CmPRR73* genes still maintained a level of the original circadian rhythm in the first day, and the amplitude of *CmPRR7* expression decreased (Fig. 9). In addition, expression of *CmPRR2* gene exhibited only a moderate circadian rhythm. On the second day, expression of *CmPRR7* showed its original circadian rhythm with an increased amplitude. Expression of *CmPRR2*, *CmPRR37*, and *CmPRR73* held a certain level of circadian rhythm with enhanced amplitude and the rhythm. In addition, after changing from the short-day to the continuous dark condition, the four *CmPRRs* maintained a distinct circadian rhythm within the 48 h tested. The amplitude and rhythm of *CmPRR2*, *CmPRR37*, and *CmPRR73* were increased.

## Discussion

In the present study, we cloned four *Chrysanthemum* PRR genes and compared their full-length protein sequences with their counterparts from other related species. Our results indicated that PRR proteins from *Chrysanthemum* are highly homologous to those from the closely related *Asteraceae* plants *H. annuus* (Fig. 1A). However, we found that all of these four CmPRRs have a relatively low homology with PRR proteins from *Arabidopsis* (Fig. 1A). For instance, CmPRR2 shares 32% identity with PRR2 (AT4G13660) from *Arabidopsis*, whereas CmPRR7 has a relatively high identity (82%) with protein AT5G02810 (PRR7). Both CmPRR37 and CmPRR73 show 46% similarity to PRR3 (AT5G60100) of *Arabidopsis*, but the similarity of them compared to PRR7 of *Arabidopsis* is 53% and 37%, respectively. In addition, we predicted motifs of the four *Chrysanthemum* PRRs. They contain the conserved Motif1 and Motif2 in the N-terminal of amino acid sequence (Fig. 1B). As expected, closely related members in the phylogenetic tree share common motifs, suggesting functional similarities among the PRR proteins within the same subtree (Fig. 1). However, the biological significance of these putative motifs remains to be elucidated as they do not have homologs when searching against Pfam and simple modular architecture research tool databases (Finn et al., 2006; Letunic & Bork, 2018).

Flower bud differentiation is the most important stage in the development of *Chrysanthemum*, which marks the transition from vegetative growth to reproductive growth. The entire process of flower bud differentiation varies by *Chrysanthemum* varieties. Previous studies by Yang et al. (2007) indicated that eight Japanese *Chrysanthemum* verities need 30–60 days to complete flower bud differentiation. In the present study, the flower bud differentiation process lasted for 64 days for Zijiao and 51 days for Aoyunbaixue (Fig. 2; Table 3). We observed that the flower bud differentiation started to speed up after the squaring stage in both Zijiao and Aoyunbaixue, which is similar to the trend observed in *Dendranthema morifolium* (Rong, 2006). Expression levels of circadian genes, including *CmGI3*, *CmCOL1*, *CmCOL5*, *CmCOL9*, *CmCOL13*, *CmCOL15*, *CmCOL16*, *CmPRR2*, *CmPRR37*, and *CmPRR37,* were higher in Aoyunbaixue, compared to that in Zijiao, suggesting that these genes may contribute to promote initiating floral meristem development. This is consistent with former studies that circadian clock genes including *PRRs* play essential roles within or close to the control of hypocotyl elongation and flowering time (Akinori et al., 2007; Ariadne et al., 2002; Eriko et al., 2002; Kaczorowski & Quail, 2003; Masaya, Takafumi & Takeshi, 2004; Makino et al., 2002; Takatoshi et al., 2007). However, other genes, including *CmGI1*, *CmGI2*, *CmCOL14*, *CmCOL20*, and *CmFT,* displayed the opposite expression pattern. These genes were highly expressed in Zijiao than that in Aoyunbaixue, suggesting their functions in inhibiting flowering (Fig. 3). Therefore, there exist two sets of circadian related genes and their interplay plays a significant role in regulating flowering time in *Chrysanthemum*. More work with knock-out mutants will help address the underlying molecular mechanisms.

During different stages of *Chrysanthemum* development, the circadian genes present different expression features in leaves. In this study, expression of *CmCOL15* was the highest at the full-blossom stage and decreased in the senescing flower stage, suggesting that it is not involved in the process of senescence. Since *CmPRR37* had a similar expression level to that of *CmGI1* and *CmCOL20*, suggesting that it plays a significant role in the regulation of *Chrysanthemum* development (Fig. 4). The expression level of tobacco *NtCO1* is significantly higher during the stage of squaring and vegetative growth, compared to the seedling stage (Lu et al., 2013). Seven *CmCOL* genes in the present study showed similar results (Fig. 4). In addition, we found that four *CmPRRs* were expressed in different tissues of Zijiao (Fig. 5). Interestingly, the *CmPRRs* had the highest expression level in flowers and stems, a moderate level in the leaves, and lowest level in the roots.

In the photoperiod pathway, plants perceive the light signal and produce the biorhythm to regulate the downstream flowering related gene expression, and then regulate the flowering process. In *C. lavandulifolium*, *ClPRR1*, *ClPRR73*, and *ClPRR37* expression peaks appeared earlier under short-day condition, compared to long-day condition (Fu, 2014). Under long-day, *SbPRR37* had expression peaks both in the morning and at night, while the peak at night disappeared under short-day condition (Murphy et al., 2011). The expression peak of *CmPRR2*, *CmPRR7*, and *CmPRR73* under long-day appeared earlier, compared to the short-day condition. However, expression of *CmPRR37* displayed the opposite pattern (Fig. 6). Previous studies indicated that the order of expression in five *Arabidopsis PRRs* is *PRR9*→*PRR7*→*PRR5*→*PRR3*→*PRR1/TOC* (Strayer et al., 2000). In comparison, five members from the *PRR* gene family were cloned from *C. lavandulifolium*, and the order of expression from morning to night in one day is *ClPRR73*/ *ClPRR37*→*ClPRR5*→*ClPRR1* (Fu, Yang & Dai, 2014). In the present study, the order of the *CmPRRs* expression peaks under long day condition appears to be like this: *CmPRR2/CmPRR73*→*CmPRR7*→*CmPRR37* (Fig. 6). Previous studies indicated that expression peaks of *SbPRR37* in both morning and night disappeared when the *Sorghum* was placed into continuous dark condition (Murphy et al., 2011). We switched Zijiao from long- or short-day conditions to continuous dark and found that expression levels of the *CmPRRs* still maintained the original circadian oscillation, but the rhythmicity and amplitude enhanced (Fig. 9). This indicates that the *CmPRRs* can maintain their circadian oscillation features, which are subject to influence by external light. When compared with the results of continuous light, expression of the *CmPRRs* has increased in both rhythm and amplitude, which may be related to the regulation of the photo responsive element in *CmPRRs* (Figs. 8 and 9). Studies have found that giving short periods of light in the dark phase under short-day, which is equivalent to long-day condition, can significantly inhibit the flowering of short-day plants (Thomas & Vince-Prue, 1997). Results from dark interrupt in rice indicated that a significant flowering delay occurred after 10 min of light is given in the dark phase (Ishikawa et al., 2005). In the present study, the changes of expression patterns of the *CmPRR*s after the night-break may impact expression of the downstream genes of the *CmPRRs* and then change the flowering process of *Chrysanthemum* (Fig. 7).

## Conclusion

In this study, four genes related to flowering time (CmPRR2C, mPRR7, CmPRR37, and CmPRR73) were isolated and cloned from *C. morifolium*. Sequences alignment and phylogenetic analysis showed that they are highly homologous with PRRs from the closely related *Asteraceae* plants *H. annuus*. In addition, conserved motifs in these PRR proteins were predicted, and we found that most of the closely related members in the phylogenetic tree contain common motif composition, which suggested the functional similarities among the PRR proteins within the same subtree. Spatio-temporal expression patterns showed that the CmPRRs were highly expressed in flower and stem tissues, and the expression levels were increasing across the *Chrysanthemum* developmental process. In addition, we found that these genes appear to be light regulated/responsive and are differentially expressed under LD and SD conditions. These CmPRRs could be involved in regulating flowering time in *C. morifolium*.

## Supplemental Information

10.7717/peerj.6420/supp-1 Supplemental Information 1 Raw data of cDNA and protein sequences of four *CmPRRs*. *CmPRR2*, *CmPRR7*, CmPRR37 and *CmPRR73* were cloned from Aoyunbaixue using the tender leaves by RT-PCR.

10.7717/peerj.6420/supp-2 Supplemental Information 2 Full length PCR amplification of *CmPRRs.*. PCR prducts were used to validate the amplificaion of four CmPRRs. Non-reverse transcribed RNA was used as the negative control. M-DNA marker; NC-negative control.

10.7717/peerj.6420/supp-3 Supplemental Information 3 Sequence logos for the conserved motifs of PRR proteins. Conserved motifs and the sequence logos were generated using the MEME search tool (http://meme-suite.org/tools/meme). Numbers on the horizontal axis represent the sequence positions in the motifs and the vertical axis represents the information content measured in bits.

## Additional Information and Declarations

### Data Availability

The following information was supplied regarding data availability: Raw data are available in the Supplemental Materials.

## References

[ref-1] Akinori M, Masakazu K, Yuko N, Takahiko K, Masaya M, Takafumi Y, Takeshi M (2007). Characterization of circadian-associated pseudo-response regulators: II. The function of PRR5 and its molecular dissection in *Arabidopsis thaliana*. Bioscience Biotechnology and Biochemistry.

[ref-2] Alabadí D, Oyama T, Yanovsky MJ, Harmon FG, Más P, Kay SA (2001). Reciprocal regulation between *TOC1* and *LHY/CCA1* within the *arabidopsis* circadian clock. Science.

[ref-3] Ariadne MP, Briana DD, Dimitrios G, Maria B, Stavroula B, Dimitrios H (2002). Blood visfatin concentrations in normal full-term pregnancies. Acta Paediatrica.

[ref-4] Atsushi O, Takako N, Li T, Takumi K, Yohei H, Katsuhiko S, Seiichi F, Tamotsu H (2012). CsFTL3, a *chrysanthemum* FLOWERING LOCUS T-likegene, is a key regulator of photoperiodic flowering in *chrysanthemums*. Journal of Experimental Botany.

[ref-5] Bailey TL, Boden M, Buske FA, Frith M, Grant CE, Clementi L, Ren J, Li WW, Noble WS (2009). MEME Suite: tools for motif discovery and searching. Nucleic Acids Research.

[ref-6] Boden SA, Cavanagh C, Cullis BR, Ramm K, Greenwood J, Jean Finnegan E, Trevaskis B, Swain SM (2015). *Ppd-1* is a key regulator of inflorescence architecture and paired spikelet development in wheat. Nature Plants.

[ref-7] Bustin SA, Benes V, Garson JA, Hellemans J, Huggett J, Kubista M, Mueller R, Nolan T, Pfaffl MW, Shipley GL (2009). The MIQE Guidelines: Minimum Information for Publication of Quantitative Real-Time PCR Experiments. Clinical Chemistry.

[ref-8] Bustin SA, Mueller R (2005). Real-time reverse transcription PCR (qRT-PCR) and its potential use in clinical diagnosis. Clinical Sciences.

[ref-9] Cha JY, Kim J, Kim TS, Zeng Q, Wang L, Sang YL, Kim WY, Somers DE (2017). GIGANTEA is a co-chaperone which facilitates maturation of ZEITLUPE in the *Arabidopsis* circadian clock. Nature Communications.

[ref-10] Eriko S, Norihito N, Takafumi Y, Takeshi MJP (2002). Aberrant expression of the *Arabidopsis* circadian-regulated *APRR5* gene belonging to the APRR1/TOC1 quintet results in early flowering and hypersensitiveness to light in early photomorphogenesis. Plant and Cell Physiology.

[ref-11] Eriksson ME, Hanano S, Southern MM, Hall A, Millar AJ (2003). Response regulator homologues have complementary, light-dependent functions in the *Arabidopsis* circadian clock. Planta.

[ref-12] Farré EM, Harmer SL, Harmon FG, Yanovsky MJ, Kay SA (2005). Overlapping and distinct roles of *PRR7* and *PRR9* in the *Arabidopsis* circadian clock. Current Biology.

[ref-13] Finn RD, Jaina M, Benjamin SBC, Sam GJ, Volker H, Timo L, Simon M, Mhairi M, Ajay K, Richard D (2006). Pfam: clans, web tools and services. Nucleic Acids Research.

[ref-14] Fu J (2014). Molecular mechanism of flower transition induced by short-day in Chrysanthemum lavandulifolium.

[ref-15] Fu J, Yang L, Dai S (2014). Conservation of *Arabidopsis thaliana* circadian clock genes in *Chrysanthemum lavandulifolium*. Plant Physiology & Biochemistry.

[ref-16] Gendron JM, Pruneda-Paz JL, Doherty CJ, Gross AM, Kang SE, Kay SA (2012). *Arabidopsis* circadian clock protein, TOC1, is a DNA-binding transcription factor. Proceedings of the National Academy of Sciences of the United States of America.

[ref-17] Gerlach D (1969). A rapid safranin-crystal violet-light green staining sequence for paraffin sections of plant materials. Stain Technology.

[ref-18] Gould PD, Locke JC, Larue C, Southern MM, Davis SJ, Hanano S, Moyle R, Milich R, Putterill J, Millar AJ, Hall A (2006). The molecular basis of temperature compensation in the *Arabidopsis* circadian clock. Plant Cell.

[ref-19] Gu C, Chen S, Liu Z, Shan H, Luo H, Guan Z, Chen F (2011). Reference gene selection for quantitative real-time PCR in *Chrysanthemum* subjected to biotic and abiotic stress. Molecular Biotechnology.

[ref-20] Hazen SP, Schultz TF, Prunedapaz JL, Borevitz JO, Ecker JR, Kay SA (2005). *LUX ARRHYTHMO* encodes a Myb domain protein essential for circadian rhythms. Proceedings of the National Academy of Sciences of the United States of America.

[ref-21] Hsu PY, Harmer SL (2014). Wheels within wheels: the plant circadian system. Trends in Plant Science.

[ref-22] Huang W, Perez-Garcia P, Pokhilko A, Millar AJ, Antoshechkin I, Riechmann JL, Mas P (2012). Mapping the core of the *Arabidopsis* circadian clock defines the network structure of the oscillator. Science.

[ref-23] Iniesta F, Testi L, Goldhamer DA, Fereres E (2008). Quantifying reductions in consumptive water use under regulated deficit irrigation in pistachio (*Pistacia vera* L.). Agricultural Water Management.

[ref-24] Ishikawa R, Tamaki S, Yokoi S, Inagaki N, Shinomura T, Takano M, Shimamoto K (2005). Suppression of the Floral Activator *Hd3a* is the principal cause of the night break effect in rice. Plant Cell.

[ref-25] Ito S, Kawamura H, Niwa Y, Nakamichi N, Yamashino T, Mizuno T (2009). A genetic study of the Arabidopsis circadian clock with reference to the *TIMING OF CAB EXPRESSION 1 (TOC1)* gene. Plant and Cell Physiology.

[ref-26] Jeanmougin F, Thompson JD, Gouy M, Higgins DG, Gibson TJ (1998). Multiple sequence alignment with Clustal X. Trends in Biochemical Sciences.

[ref-27] Kaczorowski KA, Quail PH (2003). Arabidopsis *PSEUDO-RESPONSE REGULATOR7* is a signaling intermediate in phytochrome-regulated seedling deetiolation and phasing of the circadian clock. Plant Cell.

[ref-28] Kamioka M, Takao S, Suzuki T, Taki K, Higashiyama T, Kinoshita T, Nakamichi N (2016). Direct repression of evening genes by CIRCADIAN CLOCK-ASSOCIATED1 in the Arabidopsis circadian clock. Plant Cell.

[ref-29] Kiba T, Henriques R, Sakakibara H, Chua NH (2007). Targeted degradation of PSEUDO-RESPONSE REGULATOR5 by an SCFZTL complex regulates clock function and photomorphogenesis in *Arabidopsis thaliana*. Plant Cell.

[ref-30] Koo BH, Yoo SC, Park JW, Kwon CT, Lee BD, An G, Zhang Z, Li J, Li Z, Paek NC (2013). Natural variation in *OsPRR37* regulates heading date and contributes to rice cultivation at a wide range of latitudes. Molecular Plant.

[ref-31] Kumar S, Stecher G, Tamura K (2016). MEGA7: molecular evolutionary genetics analysis Version 7.0 for bigger datasets. Molecular Biology and Evolution.

[ref-32] Letunic I, Bork P (2018). 20 years of the SMART protein domain annotation resource. Nucleic Acids Research.

[ref-33] Li G, Siddiqui H, Teng Y, Lin R, Wan XY, Li J, Lau OS, Ouyang X, Dai M, Wan J (2011). Coordinated transcriptional regulation underlying the circadian clock in *Arabidopsis*. Nature Cell Biology.

[ref-34] Liu H, Sun M, Du D, Pan H, Cheng T, Wang J, Zhang Q, Gao Y (2016). Whole-transcriptome analysis of differentially expressed genes in the ray florets and disc florets of *Chrysanthemum*
*morifolium*. BMC Genomics.

[ref-35] Lu SX, Webb CJ, Knowles SM, Kim SH, Wang Z, Tobin EM (2012). CCA1 and ELF3 interact in the control of hypocotyl length and flowering time in Arabidopsis. Plant Physiology.

[ref-64] Lu Y, Liu Y, Ren M, Mu J, Zhang X, Sun Y, Wang Z (2013). Cloning and analysis of tobacco CONSTANS homologous genes. China tobacco science.

[ref-36] Makino S, Matsushika A, Kojima M, Yamashino T, Mizuno T (2002). The APRR1/TOC1 quintet implicated in circadian rhythms of *Arabidopsis thaliana*: I. Characterization with APRR1-overexpressing plants. Plant and Cell Physiology.

[ref-37] Martin-Tryon EL, Kreps JA, Harmer SL (2007). *GIGANTEA* acts in blue light signaling and has biochemically separable roles in circadian clock and flowering time regulation. Plant Physiology.

[ref-38] Masaya M, Takafumi Y, Takeshi M (2004). Characterization of circadian-associated APRR3 pseudo-response regulator belonging to the APRR1/TOC1 quintet in *Arabidopsis thaliana*. Plant and Cell Physiology.

[ref-39] McClung CR (2006). Plant circadian rhythms. Plant Cell.

[ref-40] Michael TP, Salomé PA, Yu HJ, Spencer TR, Sharp EL, Mcpeek MA, Alonso JM, Ecker JR, Robertson McClung C (2003). Enhanced fitness conferred by naturally occurring variation in the circadian clock. Science.

[ref-41] Millar AJ, Carre IA, Strayer CA, Chua NH, Kay SA (1995). Circadian clock mutants in Arabidopsis identified by luciferase imaging. Science.

[ref-42] Mishra P, Panigrahi KC (2015). GIGANTEA—an emerging story. Frontiers in Plant Science.

[ref-43] Mizuno T, Nomoto Y, Oka H, Kitayama M, Takeuchi A, Tsubouchi M, Yamashino T (2014). Ambient temperature signal feeds into the circadian clock transcriptional circuitry through the EC night-time repressor in *Arabidopsis thaliana*. Plant and Cell Physiology.

[ref-44] Murakami M, Tago Y, Yamashino T, Mizuno T (2007). Characterization of the rice circadian clock-associated pseudo-response regulators in *Arabidopsis thaliana*. Bioscience, Biotechnology, and Biochemistry.

[ref-45] Murphy RL, Klein RR, Morishige DT, Brady JA, Rooney WL, Miller FR, Dugas DV, Klein PE, Mullet JE (2011). Coincident light and clock regulation of *pseudo-response regulator protein 37 (PRR37)* controls photoperiodic flowering in sorghum. Proceedings of the National Academy of Sciences of the United States of America.

[ref-46] Nakamichi N, Kiba T, Henriques R, Mizuno T, Chua NH, Sakakibara H (2010). PSEUDO-RESPONSE REGULATORS 9, 7, and 5 are transcriptional repressors in the *Arabidopsis* circadian clock. Plant Cell.

[ref-47] Nakamichi N, Kiba T, Kamioka M, Suzuki T, Yamashino T, Higashiyama T, Sakakibara H, Mizuno T (2012). Transcriptional repressor PRR5 directly regulates clock-output pathways. Proceedings of the National Academy of Sciences of the United States of America.

[ref-48] Nohales MA, Kay SA (2016). Molecular mechanisms at the core of the plant circadian oscillator. Nature Structural & Molecular Biology.

[ref-49] Norihito N, Masanori K, Shogo I, Takafumi Y, Takeshi M (2005). PSEUDO-RESPONSE REGULATORS, PRR9, PRR7 and PRR5, together play essential roles close to the circadian clock of *Arabidopsis thaliana*. Plant and Cell Physiology.

[ref-50] Nusinow DA, Anne H, Hamilton EE, King JJ, Takato I, Schultz TF, Farré EM, Kay SA (2012). The ELF4-ELF3-LUX complex links the circadian clock to diurnal control of hypocotyl growth. Nature.

[ref-51] Para A, Farré EM, Imaizumi T, Pruneda-Paz JL, Harmon FG, Kay SA (2007). PRR3 is a vascular regulator of TOC1 stability in the *Arabidopsis* circadian clock. Plant Cell.

[ref-52] Perales M, Más P (2007). A functional link between rhythmic changes in chromatin structure and the *Arabidopsis* biological clock. Plant Cell.

[ref-53] Plautz JD, Straume M, Stanewsky R, Jamison CF, Brandes C, Dowse HB, Hall JC, Kay SA (1997). Quantitative analysis of Drosophila period gene transcription in living animals. Journal of Biological Rhythms.

[ref-54] Prunedapaz JL, Breton G, Para A, Kay SA (2009). A functional genomics approach reveals CHE as a component of the *Arabidopsis* circadian clock. Science.

[ref-65] R Core Team (2018). R: A language and environment for statistical computing.

[ref-55] Rong Y (2006). Research on flower bud differentiation and facilitating cultivation of Autumn chrysafnthemum.

[ref-56] Salomé PA, Robertson McClung C (2005). *PSEUDO-RESPONSE REGULATOR 7* and *9* are partially redundant genes essential for the temperature responsiveness of the Arabidopsis circadian clock. Plant Cell.

[ref-57] Strayer C, Oyama T, Schultz TF, Raman R, Somers DE, Más P, Panda S, Kreps JA, Kay SA (2000). Cloning of the *Arabidopsis* clock gene *TOC1*, an autoregulatory response regulator homolog. Science.

[ref-58] Takatoshi K, Rossana H, Hitoshi S, Chua NH (2007). Targeted degradation of PSEUDO-RESPONSE REGULATOR5 by an SCFZTL complex regulates clock function and photomorphogenesis in *Arabidopsis thaliana*. Plant Cell.

[ref-59] Teixeira da Silva JA (2003). *Chrysanthemum*: advances in tissue culture, cryopreservation, postharvest technology, genetics and transgenic biotechnology. Biotechnology Advances.

[ref-60] Thomas B, Vince-Prue D (1997). Photoperiodism in plants.

[ref-61] Turner A, Beales J, Faure S, Dunford RP, Laurie DA (2005). The pseudo-response regulator *Ppd-H1* provides adaptation to photoperiod in barley. Science.

[ref-62] Yang N, Wei-ming G, Fa-di C, Wei-min F (2007). Effects of photoperiod on floral bud differentiation and flowering of *Chrysanthemum morifolium* Ramat “Jinba”. Acta Horticulturae Sinica.

[ref-63] Yoko Y, Eriko S, Tomo S, Norihito N, Shusei S, Tomohiko K, Satoshi T, Akira N, Takafumi Y, Takeshi M (2003). Comparative genetic studies on the *APRR5* and *APRR7* genes belonging to the APRR1/TOC1 quintet implicated in circadian rhythm, control of flowering time, and early photomorphogenesis. Plant and Cell Physiology.

