# Supplementary material for: Expression analysis of four pseudo-response regulator (PRR) genes in Chrysanthemum morifolium under different photoperiods
Source: PeerJ. 2019 Feb 19;7:e6420. doi: 10.7717/peerj.6420 (PMC6385685; doi:10.7717/peerj.6420)
Supplement: Supplemental Information 1 — CmPRR2, CmPRR7, CmPRR37 and CmPRR73 were cloned from Aoyunbaixue using the tender leaves by RT-PCR. [file peerj-07-6420-s001.doc]

**>RRR2_cDNA**

ATGGTTTGCACTGCGAACGACTTATTGGAGTGGAAAGACTTTCCAAAGGGTCTTAGAGTCTTGCTACTTGATGAAGATATCAATTCTGCAAATGAAATAAAATCAAAACTTGAGGAAATGGATTATGTTGTCACGATGTTCTATACTGCGAATGAAGCATTGTCAGCCGTTTCAGATAAATCTAGAAGCTTCCATGTAGCAATTGTAGAGGTGAGGGCAGACAACGAAGATGGAAGGTTTAAGTTTCTTGAAAGTGCAAAAGAGTTGCCTACAATCATGACTTCAAATGTCAATTGCATTAGTACCATAATGAAGTGCATAGCGCTCGGTGCGGTCGAATTCCTCGAGAAACCACTTTCAGAAGATAAACTTAGAAACATATGGCAACATGTGGCTCACAAGGCGTTTAATGCGGGACCAAAGGATCAAGCTGAAGCATTGAAATCTAAGAATGCATCGGCTCCAGAGGTTCATTTGAAAACAGATATAAAGGTAGAGGTTAGTCAAAATCAAGAACATAATGTAGAGGTATCTGTTGAAGGTGATAAATATCTGGCTCCATCAACCCCCCAATTGAAGCATGGAATTAGGCTAGTTGATAATGGTGACTGTCAAGATCATGAGCATGATCATGATCAAGTTCAAACTAATCTCTCGACCGAGAAAGAGTGTGTAGATCATGATGATCCATCCAAATTTGTCGAAACTATTTGTAAAGGTTTAGTTGTCTATACTACTGTTATTACCGAGACTGCTTCATCCAAGAGTTTGGAGGAGGGTATTCTCAAGCCGTTTACTAGTGAATGCAGACCTGATCCTAAAGATGATGGTAAGAAGCCAAATGAAGTATCTGAGAATCCTAGTTCACGTGCTACTAAATCAAGTCGAAGAAAGATCAAGGTAGATTGGACTTCTGAACTACACAAGAAATTCGTACAAGCTGTAGAGCAACTCGGTGTTGATCAAGCAACACCTTCTCGTATACTTGAACTTATGAATGTAGAGGGTTTGACTCGGCATAATGTAGCAAGTCATCTGCAGCAGAAATATAGATTGCAACGAAGACATATATTGCCAAAAGAAAGTGCGCGAAAATGGCCACAAACCAGACATTCAACACCAAGAAACTACTATTCGCCGAGGCCCATAATGGCTTACCCTCCATATCATTCTAATCACACCCTTCCAGCCAACCAAGTGTATCCTGCTTGGCCACCTCCGACCAATTATGCTCCACCACCTCGGATGTGGAGCCCACCGTATTATCAAGCATGGCATTCTTCCGAAAATTGGATGTGGAATCCTTATCCAGGGGTGCAAGCCGAAGCATGGGGCTGCCCGACTTTACAGGCACATCCATCTTTTCCTCAAAATGCTTCTCAATATCATAGTAGGAATTCAATGCAGAGTGGCCAGACCATGCCAAAGAACTCACTCGACAATTATCCGGGAGATGAAGTCATTGACAAGGTTGTAAAGGAAGCAATAAATAAGCCATGGCTACCCTTGCCTTTAGGCCCCAAACCTCCATCAACGGAGAGCGTCATTTCTGAGCTCTCCAGACAAGGCATTTCCACGGTTCCTCCTAGAATCAGCAGCTCTCGTTAA

**>RRR2_protein**

MVCTANDLLEWKDFPKGLRVLLLDEDINSANEIKSKLEEMDYVVTMFYTANEALSAVSDKSRSFHVAIVEVRADNEDGRFKFLESAKELPTIMTSNVNCISTIMKCIALGAVEFLEKPLSEDKLRNIWQHVAHKAFNAGPKDQAEALKSKNASAPEVHLKTDIKVEVSQNQEHNVEVSVEGDKYLAPSTPQLKHGIRLVDNGDCQDHEHDHDQVQTNLSTEKECVDHDDPSKFVETICKGLVVYTTVITETASSKSLEEGILKPFTSECRPDPKDDGKKPNEVSENPSSRATKSSRRKIKVDWTSELHKKFVQAVEQLGVDQATPSRILELMNVEGLTRHNVASHLQQKYRLQRRHILPKESARKWPQTRHSTPRNYYSPRPIMAYPPYHSNHTLPANQVYPAWPPPTNYAPPPRMWSPPYYQAWHSSENWMWNPYPGVQAEAWGCPTLQAHPSFPQNASQYHSRNSMQSGQTMPKNSLDNYPGDEVIDKVVKEAINKPWLPLPLGPKPPSTESVISELSRQGISTVPPRISSSR

**>RRR7_cDNA**

ATGAGGAGTGTTGGTGTAAACACTAATGTTCCGACAACTAAAGGGTTGGCGGAACTTAACCATCATACTCGTGTCGAGCTAAAAGAAGTAAGAAATGGAGCCGGGTTTGATGGTCGAGGCTTGTCTGACGAAGATGAGTCAAGGATTAATGAAGATGTTAGGCACATAGGGTCGGTCCAGATGCTTGATGGTCTCCATAGACAACATCAAGGGTCGCTTGTTCAGTGGGAGCGGTTTTTCCCTAGTAAGTCGCTTAAGGTTCTTTTGGTTGAAGATGATGATTCGACTCGTCACGTTGTCAGTGCTTTGCTCCGTAATTGCGGCTATGAAGTTATGGCGGTGGCGAACGGTCTAGAAGCATGGAAAGTGTTGATTGATGTCAGCAAACAAATTGATCTTGTTCTGACCGAGGTAGTCATGCCATATTTATCGGGTATTGGTCTTTTGACCAAGATCATGAACAACGTTACTCGCAAGAATACTCCAGTGATAATGATGTCATCCGATGACTCAATGGGTATAGTCTTTAATTGTTTATCCAAAGGTGCAGTTGACTTTTTAGTCAAGCCTATTCGAAAGAATGAGCTAAAAAACATCTGGCAGCATGTGTGGAGGAAGTGTCACAGTGTGAGTGAATTTAGTTTTATGGTTTTGTGTATGATTAAAAACGGACCAGTTTTGGACTTCGGCTTCAGGTGTGTTCTGATTTCGTATGTTTTCTTATCGTGGGGTGGAGTGTACTGGATTGGAGCTAACTACTACTTCTCCAATATGCTCATCTGTCCATGTGAAATTTTGAAAAAACAATCAGTAAGAAACGAAATTCTTTTTTCTGTTCTGCTTGAATTTCTCGTATTCTCAGCAGTCGAGTGGGAGCGGGAGTGGGAGGGAAAGTGGTATACGGGAGGAAAAAGCTACAAAATCAAGAAGCATGGAAGAGTCGGAGGATGGCAGGGACAACAGGGACGAAGAGGAGGATGTAAGCATAGAGTTGAACGCGAAAGGTGGAAGCGATAA

**>RRR7_protein**

MRSVGVNTNVPTTKGLAELNHHTRVELKEVRNGAGFDGRGLSDEDESRINEDVRHIGSVQMLDGLHRQHQGSLVQWERFFPSKSLKVLLVEDDDSTRHVVSALLRNCGYEVMAVANGLEAWKVLIDVSKQIDLVLTEVVMPYLSGIGLLTKIMNNVTRKNTPVIMMSSDDSMGIVFNCLSKGAVDFLVKPIRKNELKNIWQHVWRKCHSVSEFSFMVLCMIKNGPVLDFGFRCVLISYVFLSWGGVYWIGANYYFSNMLICPCEILKKQSVRNEILFSVLLEFLVFSAVEWEREWEGKWYTGGKSYKIKKHGRVGGWQGQQGRRGGCKHRVERERWKR

**>RRR37_cDNA**

ATGAAGAGTGTTGGAGTGAGTACTAGTGTTCCGGTAACTAATGGATCCGCGGAACTTAATTATCATAAACGAAATGAGTTGAAAGAAGTGAGAGACGGAGGTGCGTTTAATGGTCAAAGGTTGTCTGATGAAGATGAGTCTAGGATTAATGAGGATGTTAACGGTGGTAGGAAGATAGAGTTGGTTGTATCGCAGGATGTTGTTAAACGAACTCATCCGCAAGCTCAAGGGCCGTTGGTTCAATGGGAGAGGTTTTTGCCTGTTAGGTCTTTGAAAGTTTTACTAGTGGAAGATGACGATTGTACTCGACACGTTGTTAGTGCGCTGCTACGTAATTGCAGCTATGAAGTTACCGCTGTTGCAAATGGTCTAGAAGCATGGAAGGTACTGGTTGATCTCAACAAACAAATTGACCTTGTTTTAACTGAAGTGGTCATGCCATTTCTATCTGGAATCGGTCTTTTATCCAAGATCATGAACCACTTACCACGCAAAAATACGCCAGTGATTATGATGTCATCTGATGATTCTATGGGTATAGTCTTTAACTGTTTATCCAAAGGTGCAGTTGACTTTTTAGTCAAACCCATTCGAAAGAATGAGCTAAAAAACCTCTGGCAGCATGTTTGGAGGAAATGTCACAGTTCTAGCGGTAGTGAGAGCGAGAGTAGGATACATGCTCGTAAAACTGCTAAGGCGAGAAGCATTGAAGTCTCGGATGATGACAATGATAGCAGTGATGAGGATGATGACAGGAGCATAGGCCTGGCCGCGAAGGATGGAAGTGATAATGGAAGTGGCACTCAGAGTTCTTGGTCAAGAAGAGCAGTAGAAGTTGAAAGTGCAAAGTATTCGTGGGATCAGTTAGCGGGGCCCTCTCAAAGCACTTGTGCCCAGGGTATCCATGCAAGACCAGATAAAGTGAACACAATAGAACATAATGCTGGAGAAGATGATAACATGGATGCAATTGCAATGGGGAAAGATTTGGAAATTGGAGTCCCTAGAAATTCAGACAAAATTATCGAATTAAATACGAATGAAATGGGTGAACCAAAGACCGCTAATAATGTAATGATTGCGAAAACAAATGGATCAGTCAACAGTAAGGGATCACCTGCCCTAGAACTCAGTTTGAAGAGACCACGTGACTTGGAGGATACTGATATGAGCACTCAAAAGCGAAATGTTATTAGACAGTCGGATCTTTCAGCCTTCTCAAGGTATAACAATACCACATCAAATGCCAATCAAGCTACAACTGGAAATGTGGGCAGCTGTTCTCCAGTAGATGTCACTATCAGTTCAGAAGCCGCAAAACTCATGAACATACAATCTAACTCAAATGGAGCCCCAAATCAACGTTCTAATGGCAGCAGCACTCACAACAACGATATGGGCTCGAGCACTAACAATGCATTCTCCAAACCAGAACCTCCTGCAGATGACAAGACCATTCCTCCTGGTCCTTCTGATGGTGACAAGGCACAGTTTCAAGTCCGCCACCATCATCACCATTATCACCACCACCACCATCATCATGTCCACAAAACACAACAACAGCAACAACAACAACAACAACAACAACAAACAGATAACCAAGATGACGGATCTTCAGGAAACTTGGTATCTAACACTTTGGCTGCACCTGTTGAAGGAAATGCTGCTAATTATGGGAGTGCATCTGGAAGTAATAATAAAAGTAATGGAGAGAATGGCAGCAGTGGCCAAAAGGGTAACAGCTATACAGCCGTAGCCGAAGGGGGTGATAATGGAGTTGTTGAAAAGGTTAAATCTGGAAATGGAAGCGGAAGCGGAAGTGGAAGGGGTAGTGAGGTTGATCAAGACCGATTAGCGCAGAGAGAAGCTGCACTTATCAAGTTCCGCCAGAAGAGGAAAGAAAGATGCTTTGAGAAGAAGGTACGATATCAAAGCAGGAAAAAGCTGGCAGAACAGAGACCACGTGTTCGCGGACAATTTGTAAGACAAGGGGTGAATGGGAAGGATGCTGATTCCTGA

**>RRR37_protein**

MKSVGVSTSVPVTNGSAELNYHKRNELKEVRDGGAFNGQRLSDEDESRINEDVNGGRKIELVVSQDVVKRTHPQAQGPLVQWERFLPVRSLKVLLVEDDDCTRHVVSALLRNCSYEVTAVANGLEAWKVLVDLNKQIDLVLTEVVMPFLSGIGLLSKIMNHLPRKNTPVIMMSSDDSMGIVFNCLSKGAVDFLVKPIRKNELKNLWQHVWRKCHSSSGSESESRIHARKTAKARSIEVSDDDNDSSDEDDDRSIGLAAKDGSDNGSGTQSSWSRRAVEVESAKYSWDQLAGPSQSTCAQGIHARPDKVNTIEHNAGEDDNMDAIAMGKDLEIGVPRNSDKIIELNTNEMGEPKTANNVMIAKTNGSVNSKGSPALELSLKRPRDLEDTDMSTQKRNVIRQSDLSAFSRYNNTTSNANQATTGNVGSCSPVDVTISSEAAKLMNIQSNSNGAPNQRSNGSSTHNNDMGSSTNNAFSKPEPPADDKTIPPGPSDGDKAQFQVRHHHHHYHHHHHHHVHKTQQQQQQQQQQQQTDNQDDGSSGNLVSNTLAAPVEGNAANYGSASGSNNKSNGENGSSGQKGNSYTAVAEGGDNGVVEKVKSGNGSGSGSGRGSEVDQDRLAQREAALIKFRQKRKERCFEKKVRYQSRKKLAEQRPRVRGQFVRQGVNGKDADS

**>RRR73_cDNA**

ATGACTAGTAGCAGCAGAGGAAGGAGCAAGGGTTCTAGACAGGTGAATGGTCGTGTGAAAGCTGAGATTCGGGGAGTTGTGAATGGATTTGCGAGTAATGGATCCAGTTCACGAGGAGATCATGAAAACGTGCAGGATGGTGGCAACAAGGTGGTTGTGACAGGGAACGGAGTTCCTCAAGCACCTCAGCAACTGCCTCAAGGGTCATCGGTCCAATGGGAGAAGTTTCTTCATGTTAGATCCATTAAGGTCATGTTGGTTGAAGACGATGATTGTACACGTCACATTGTTACTGCTTTGCTTCGCAACTGTAACTATGAAGTTATTGAAGCAGCCAATGGATTTCAAGCTTGGAAGATATTACAAAATCTATCCAATCACATTGACCTTGTTTTAACCGAAGTAATCATGCCTTCTCTTTCAGGAATCGGTCTTTTATGCAAGATTATGAGCCACAAAACACGAAAGAATGTTCCTGTGATTATGATGTCTTCTCATGATTCAATGGGTTTGGTTTTTAAGTGTTTATCAAAAGGTGCAGTAGACTTTTTAGTGAAACCTATCCGGAAAAATGAGCTTAAAAATCTTTGGCAGCATGTGTGGAGGAGGTGTCACAGTTCAAGTGGGAGTGGGAGTGAAAGTGGCACACAGGCCCAAAAATCCGTAAACTCAAAAAGCAATTTAAGGTATGATAACCGCTGCAGCAGAGATGGGGATGACGATGAGAGCACCGATGGTGGTAGCGATGATGGTAGTGACACTCAGAGCTCTTGGACAAAACAAGCTGTTGACTGCGAGAGCTCAGAAGCTGCATCTCCTTGTGATAACATAGCTGAGCATCCAGACAGCACTTGTGGCCTCGTTATCCGTCCTGTAAGGGACTACAATGGTCAGAGTGAACAACCCTTTTCAGACGATGGTGGAAAGGCTAAAGAGATTGCAATTAGCAGATCAAGAAACTCAGAAATGCAAGTTGAGTTTCCAATTGAGGCCCCTACGAAAGATAATGGTATTAAACAAAACTCTTACATGGAAAATGGGAAAACTGATAGTCAAGGAGTTCCCGAAAAGATTATGGAGGAAAATATCAAGGTTATCGATGATTCCAATGGAGTGATAACTGGTGAGCTTGGTTTAAAGAGGCCCCGTGCAACTGATAATGATGGGGGAGAAGTTCAAAATGTCTGTCATATTTTAAGACATTCAGAGCTCTCAGCCTTCACAAGGTATAAATCAAAAACAAATGCTGCAACTGTGACAACTGGAATCACTGCCAGCTGTTCCCAACCTGCTAATATACCTAACATTGTAAAAAATGAATCCAAGCGTGACACACATTCAGATGGATATCTTATTTATCAAGGCTCAAGTGAGCAAGTCATACCAAGAAAAGATGATGATGACATGGCACCTCAGGAGCTTCGTATTCAACACATTCATCATCATCACCATGTTCATCATTACCATAACATAGATACAGACCAGCCATTATCCAATCAGAATGATTTTGGGCTAAACGATTTGGGCGCGGATGCTCCACACTGCGGGTCATCAAATATAATGGGCGGGCCTGTTGAAGGTAACCTTGAAAATTATAGCTTAAACAAAAGTGGCTCTGGCAGCAAGCATGGAAGCTGCAACGTGCAGAATGGGAGTAACACTGCAGTGAATATCGAAGGTACAAATGTAGAAAGCAATGTTAGTATAGTTGAGAAAAGTGGAAGTGGTGGCGATGGTAGTGGAAATAATAACCATAAATCTGCACAGAGAGAAGCAGCTTTGACTAAGTTCCGAAAAAAGAGAGAAGTGAGATGCTTCCAAAAGAAGGTGCGGTATCAAAACAGAAAGAAACTGGCAGAACAAAGGCCACGGGTGCGCGGACAATTTGTGAAGGGAACTAGTCGTGACGGTTCCAGTTCCGCTAATGCTGCTGATGTCTAG

**>RRR73_protein**

MTSSSRGRSKGSRQVNGRVKAEIRGVVNGFASNGSSSRGDHENVQDGGNKVVVTGNGVPQAPQQLPQGSSVQWEKFLHVRSIKVMLVEDDDCTRHIVTALLRNCNYEVIEAANGFQAWKILQNLSNHIDLVLTEVIMPSLSGIGLLCKIMSHKTRKNVPVIMMSSHDSMGLVFKCLSKGAVDFLVKPIRKNELKNLWQHVWRRCHSSSGSGSESGTQAQKSVNSKSNLRYDNRCSRDGDDDESTDGGSDDGSDTQSSWTKQAVDCESSEAASPCDNIAEHPDSTCGLVIRPVRDYNGQSEQPFSDDGGKAKEIAISRSRNSEMQVEFPIEAPTKDNGIKQNSYMENGKTDSQGVPEKIMEENIKVIDDSNGVITGELGLKRPRATDNDGGEVQNVCHILRHSELSAFTRYKSKTNAATVTTGITASCSQPANIPNIVKNESKRDTHSDGYLIYQGSSEQVIPRKDDDDMAPQELRIQHIHHHHHVHHYHNIDTDQPLSNQNDFGLNDLGADAPHCGSSNIMGGPVEGNLENYSLNKSGSGSKHGSCNVQNGSNTAVNIEGTNVESNVSIVEKSGSGGDGSGNNNHKSAQREAALTKFRKKREVRCFQKKVRYQNRKKLAEQRPRVRGQFVKGTSRDGSSSANAADV
