# Supplementary figures and images for: Expression analysis of four pseudo-response regulator (PRR) genes in Chrysanthemum morifolium under different photoperiods
Source: PeerJ. 2019 Feb 19;7:e6420. doi: 10.7717/peerj.6420 (PMC6385685; doi:10.7717/peerj.6420)

M

*CmPRR2*

*CmPRR7*

*CmPRR37*

*CmPRR73*

NC

2000bp

1000bp

750bp

500bp

250bp

100bp

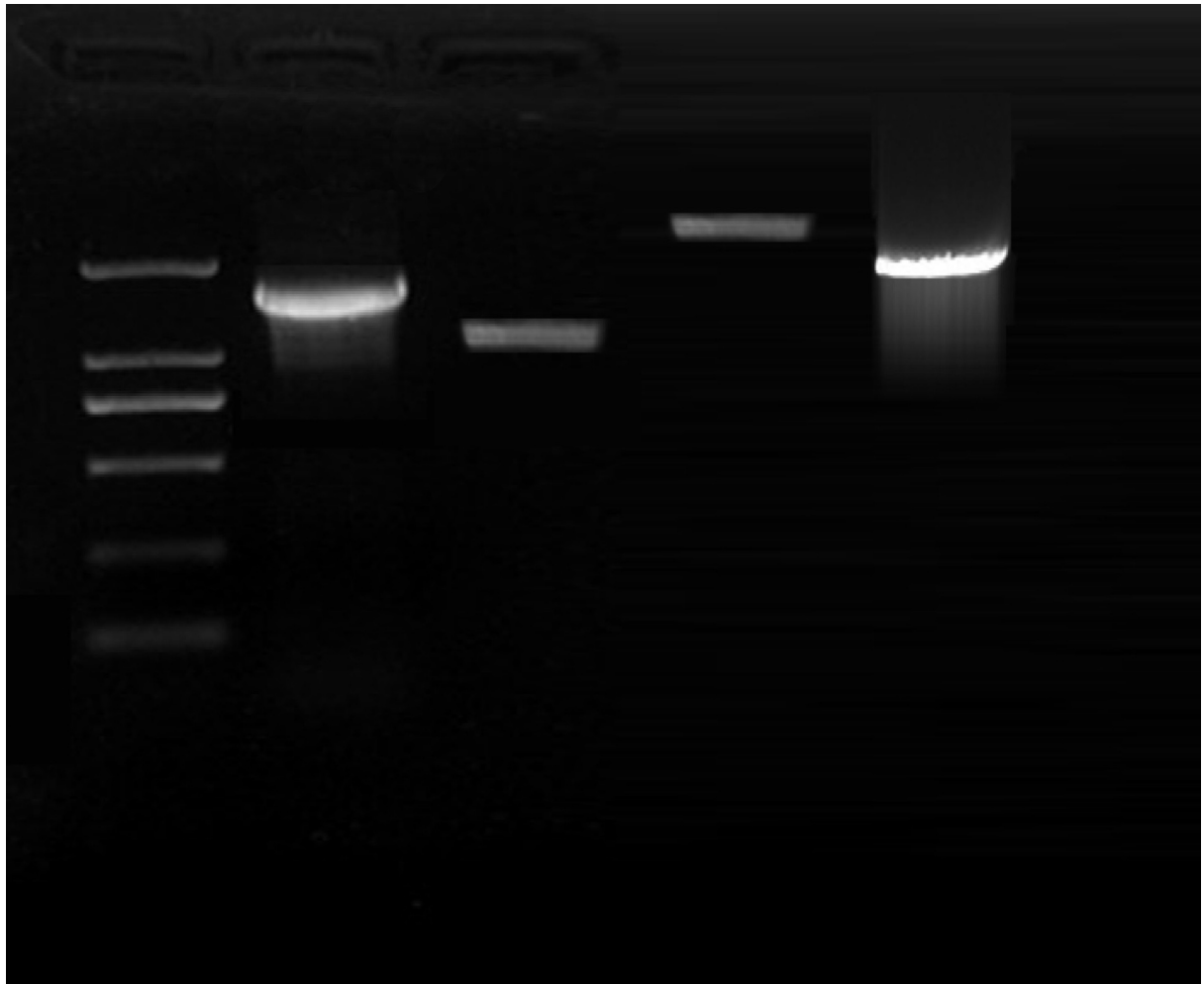

Supplement: Supplemental Information 2 — PCR prducts were used to validate the amplificaion of four CmPRRs. Non-reverse transcribed RNA was used as the negative control. M-DNA marker; NC-negative control. [file peerj-07-6420-s002.pdf]
